# Non-surgical treatment of stage 4A retinopathy of prematurity

**DOI:** 10.1186/s12886-024-03434-5

**Published:** 2024-04-19

**Authors:** Ehsan Namvar, Alireza Attar

**Affiliations:** Poostchi Ophthalmology Research Center, Department of Ophthalmology, School of Medicine, Shiraz University of Medical Sciences, Shiraz, Iran

**Keywords:** Retinopathy of prematurity, Stage 4A ROP, Anti-vascular endothelial growth factor (VEGF)

## Abstract

**Background:**

Retinopathy of prematurity (ROP) is a major cause of visual impairment in premature infants, often requiring surgical interventions in advanced stages. This retrospective case series study investigates non-surgical management for Stage 4A ROP, specifically the use of combined laser therapy and intravitreal anti-vascular endothelial growth factor (VEGF) injections.

**Methods:**

Ten eyes from five infants with Stage 4A ROP were treated with a combined laser and anti-VEGF approach. Comprehensive follow-up examinations were conducted to evaluate the treatment outcomes.

**Results:**

The study demonstrated successful retinal attachment without complications, showcasing the efficacy and safety of this non-surgical method. A comparison with surgical interventions highlighted the potential benefits in terms of reduced adverse effects.

**Discussion:**

This combined treatment emerges as a promising first-choice option for Stage 4A ROP, offering rapid regression without surgical intervention, particularly in early stages. However, larger randomized clinical trials are necessary to validate these findings and establish definitive guidelines for managing this complex condition.

**Conclusion:**

Combined laser and anti-VEGF therapy proved to be an effective and safe non-surgical approach for Stage 4A ROP, with the potential to reduce the need for surgery, especially in its early presentation. Further research is required to confirm these findings and provide comprehensive recommendations for clinical practice.

## Background

Retinopathy of prematurity (ROP) stands as the primary cause of visual impairment among infants globally [1]. This vasoproliferative disorder primarily impacts preterm and underweight newborns [2]. ROP stage 4 was defined by International Classification of Retinopathy of Prematurity (ICROP) as ROP with extrafoveal (4A) or foveal (4B) partial retinal detachment (RD) [3]. Moreover, the amount of VEGF in the vitreous body is high in ROP stage 4 [4]. Several studies reported good anatomical and visual outcomes for intravitreal anti-VEGFs in ROP stage 4 [4, 5]. The conventional treatment for high-risk pre-threshold retinopathy of prematurity (ROP) involves traditional laser therapy targeting the non-vascularized retina, eliminating cells producing Vascular Endothelial Growth Factor (VEGF) [6]. However, there is a recent shift towards adopting anti-VEGF agents [6]. Laser photocoagulation has a success rate of 80–85%, aiming to stop disease progression [7, 8]. Surgical intervention is essential for stages 4B and 5, with lens sparing vitrectomy (LSV) showing promise. Despite advancements, surgical interventions, including LSV, have complications [9]. Treating stage 4A aims to prevent progression to stage 4B or 5, requiring timely interventions for disease control and vision preservation [6]. In this study, we focus on patients who had ROP stage 4a but were managed using non-surgical methods including combined laser and intravitreal anti-VEGF.

## Materials and methods

**Fig. 1:**
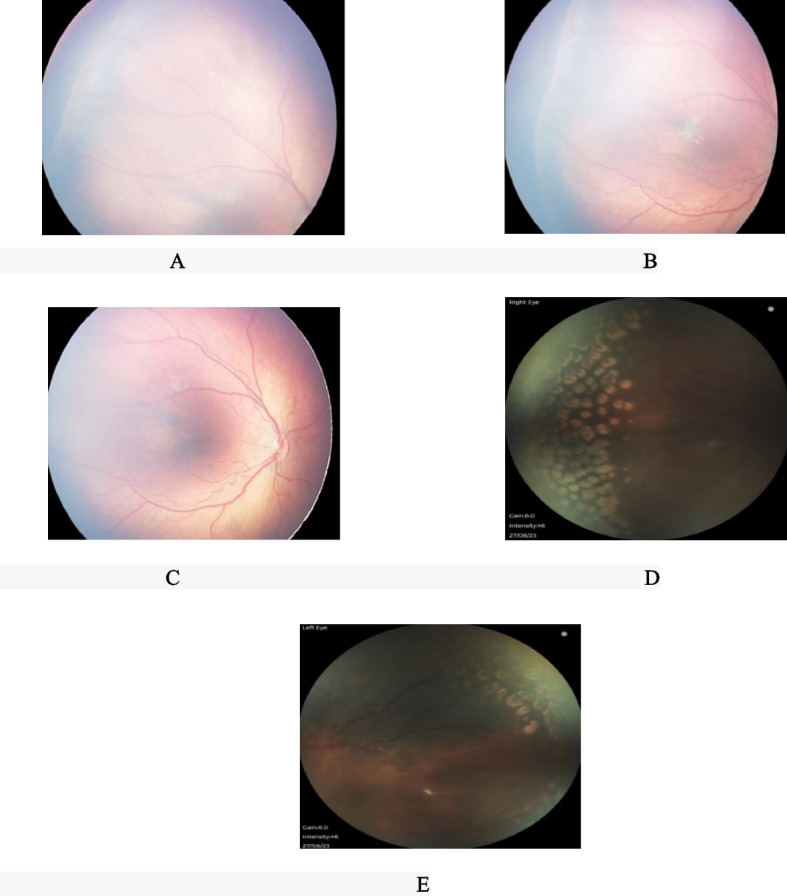
**A**, **B**, **C** Color fundus photographs before treatment with intravitreal injections and laser, showed arterial tortuosity and venous dilation of the posterior vessels, there was a ridge with retinal detachment in the peripheral retina and attached macula. **D**, **E** Color fundus photographs after treatment showed 360º degrees laser in the peripheral retina and attached retina

This is a retrospective case series study. All medical records of patients with ROP stage 4a were evaluated. The research protocol received approval from the institutional ethics committee and adhered to the principles of the Declaration of Helsinki. In this study, a total of 10 eyes from 5 infants with stage 4 ROP who referred to Poostchi Eye Clinic were enrolled in 2023. All patients initially presented as primary referrals without any prior therapeutic interventions. In the examination, we observed arterial tortuosity and venous dilation of the posterior vessels, as well as a ridge in the peripheral retina with associated retinal detachment. Fortunately, the macula remains attached (Fig. 1A, B, C). Subsequently, all of them underwent simultaneous laser photocoagulation and intravitreal anti-VEGF injections by vitreoretinal fellowship. All steps and procedures were comprehensively elucidated in the procedure section. Follow-up examinations were done on days 1, 3, 5, and 7 after treatment and then every week for 1 month, followed by every 2 weeks until confirmed attachment of the retina and no active ROP, thereafter every 4 weeks till 1 year. During the follow-up assessments, it was observed that the retinas of all patients remained attached (Fig. 1D, E), and there was no need for vitrectomy. Demographic and medical Data, age at treatment, stage and location of ROP and the width of partial retinal detachment as clock hour were recorded.

### Procedure

After pupillary dilatation with 1% tropicamide, indirect retinal laser photocoagulation with near confluent laser spots was performed. Subsequently, preparation of the eyelids and conjunctiva was conducted using 10% povidone iodine, followed by the placement of a lid speculum. After a 3-min interval, a dosage of 0.625 mg (0.025 ml) of bevacizumab was administered into the vitreous cavity utilizing a 30-gauge needle. The needle was inserted through the supratemporal quadrant of the pars plana, positioned 1.5mm posterior to the limbus. The affected eye was treated with topical antibiotics, including gentamicin ophthalmic drops (3mg/ml) three times daily, and betamethasone ophthalmic drops (0.10%) four times daily.

### Evaluation of surgical outcomes

The evaluation of anatomical results was performed. This assessment involved binocular ophthalmoscopy and the capture of fundus photographs using phoenix digital fundus camera during subsequent follow-up appointments. During follow-up, changes in retinal detachment, stage and extent of ROP, retinal vessels, plus disease and macular contour were monitored. Inadequate regression after treatment was defined as persistent ROP. Retinal vascularization arrest with new demarcation line, ridge, or RD was defined as recurrent ROP [10]. In patients with persistent ROP or recurrent ROP, retreatment with either intravitreal injection of anti-VEGFs or surgery including lens sparing core vitrectomy for removing traction may be considered. In addition, visual and refractive outcomes and ocular alignment were evaluated. Intravitreal complications including endophthalmitis, cataract, vitreous hemorrhage, and also major systemic conditions were recorded.

## Results

The baseline characteristics of all patients are summarized in Table 1.

**Table 1:** Summary of variables statistics in the study

Variable	Mean	Standard deviation
Birth weight(gr)	1399	1220.82
Age (week)	30.6	0.94
Duration of NICU stay(day)	32.6	31.34
Duration of O2 consumption (day)	22	20.21
Age at the time of laser and injection (week)	39.4	3.24

In this study, we had 5 infants, 4 of whom were female and 1 was male. Among them, one was a twin. Except for one infant who developed sepsis and received blood transfusion, the others had no specific past medical history. All patients were in stage 4a zone2 except one who was in stage 4a zone1 (Table 2).

**Table 2:** Severity, location and extent of ROP in patients

Patients	Stage	Zone	Extent of retinal detachment
Patient 1	4a	1	5 clock hours
Patient 2	4a	2	4 clock hours
Patient 3	4a	2	4 clock hours
Patient 4	4a	2	4 clock hours
Patient 5	4a	2	4 clock hours

Neonatal Demographics and Medical Parameters Dataset of all patients are shown in Table 3.

**Table 3:** Neonatal patient demographics and medical parameters dataset

Patients	Sex	Birth weight (gr)	Age (week)	Duration of NICU stay(day)	Duration of O2 consumption (day)	Age at the time of laser and injection (week)	Associated risk factors
Patient 1	Female	890	29	92	70	44	O2 consumption, septicemia, Blood transfusion
Patient 2	Female	1500	31	21	7	39	O2 consumption, respiratory distress syndrome
Patient 3	Male	1745	30	12	5	35	O2 consumption, respiratory distress syndrome
Patient 4	Female	2060	32	14	7	42	O2 consumption, Blood transfusion
Patient 5	Female	1800	31	24	21	37	O2 consumption, respiratory distress syndrome

The study variables included birth weight (1399g, 1220.82), age (30.6 weeks, 0.94), NICU stay (32.6 days, 31.34), O2 usage (22 days, 20.21), and laser/injection age (39.4 weeks, 3.24. No infant experienced complications after the surgery, and all infants’ retinas were successfully attached and inactive. No reactivation or retreatment was reported. Macula of all patients was normal without any dragging or distortion after 1 year. No ocular side effects including cataract, endophthalmitis, vitreous hemorrhage was reported. One year after laser and anti-VEGF injections, patients underwent refraction. Table 4 uniformly reports their refraction and visual acuity, all recorded as CSM.

**Table 4:** Refraction and visual acuity of the patients after laser and anti-VEGF injection

Patients	Cyclo-refraction		Visual acuity
Patient 1	OD: -0/5	OS: -1	CSM
Patient 2	OD: plano	OS:-O/5	CSM
Patients 3	OD: -1/25	OS: -1/25	CSM
Patients 4	OD: -1	OS: -1/5	CSM
Patients 5	OD: +1	OS: +1	CSM

## Discussion

Although vitrectomy or scleral buckling is suggested for stage 4 ROP, these treatments may have many adverse effects including endophthalmitis, iatrogenic retinal break, cataract, and delayed-onset intraocular pressure elevation after vitrectomy [11–13] and anisometropia, high myopia, and the need for removal of the buckle after scleral buckling [14, 15]. According to the ETROP study, vitrectomy in ROP stage 4a is not as successful as other case series [12]. Regression rate in patients with stage 4a ROP who were treated with laser alone was 55.6% and 76.9% in those who were treated with anti-VEGF intravitreal injection alone [9]. Therefore, combined laser and anti-VEGF intravitreal injection might have more effects, similar to our study. A meta-analysis study reported no statistically significant differences between anti-VEGF treatment and laser treatment in the regression rate. However, anti-VEGF treatment had a higher recurrence and retreatment rate. In addition, safety outcomes were similar between the two treatments. IVB was associated with fewer surgical interventions and better refractive outcomes [16]. Even if vitrectomy is required, anti-VEGFs could reduce intraoperative bleeding, surgery time, endodiathermy during surgery, postoperative complications and increase percentage of lens preservation, anatomical reattachment and vision recovery [17]. However, anti-VEGFs may increase the risk of proliferative membrane contraction and consequently may exacerbate retinal detachment [18–20]. In addition, laser photocoagulation does not take effect immediately as Paulus YM. Et al study represented that retinal scar stabilizes about 1 month after laser at about 35% of the initial lesion size and at the same moment retinal gliosis occurs in all retinal layers, therefore in the meantime retinal detachment may be aggravated [21]. However, laser and bevacizumab combination therapy is safe and contributes to more rapid regression compared with laser monotherapy [22]. This method decreases rate of retinal detachment compared with anti-VEGF monotherapy. Over all it seems to be more beneficial in stage 4a ROP, like our study, than laser or intravitreal anti-VEGF monotherapy. As mentioned in Sukgen et al.’s study [9], Cases with less than 6 clock hours in stage 4a are more responsive to non-surgical treatments, similar to our cases. Combined laser and anti-VEGF injection seems to be the best first choice treatment in ROP stage 4a. In the follow-up, if any progression was seen, treatment was immediately converted to surgical treatment. If no progression was seen, follow-up continued till complete regression of ROP.

The present study is subject to several limitations, encompassing its retrospective design, a relatively modest sample size, a truncated follow-up period, and the absence of a control group. To address and enhance these limitations, it is recommended that future investigations adopt a prospective approach, encompassing a larger cohort, implementation of a control group, and an extended follow-up duration.

## Conclusion

This study demonstrates the efficacy of combined treatment in effectively controlling enrolled cases, resulting in stable conditions after 1 year of close follow-up. These findings suggest that the combined treatment method is effective for stage 4A ROP cases. However, it should be noted that this study had a limited sample size and a relatively short follow-up period. Therefore, further analysis is warranted to increase the number of cases, extend the duration of follow-up, or even include similar cases from different institutions to enhance the reliability of the study conclusions.

## Data Availability

The datasets used and analyzed during the current study are available from the corresponding author on reasonable request.
